# Recent progress in stem cell and immune cell-based interventions for aging and age-related disorders

**DOI:** 10.3389/fragi.2025.1638168

**Published:** 2025-07-22

**Authors:** Li He, Donglei Han, Fenfen Zong, Yan Zhang, Zhongyang Han, Zenghui Xu

**Affiliations:** ^1^ Henan Cell Therapy Group Co., Ltd, Zhengzhou, China; ^2^ Shanghai Cell Therapy Group Co., Ltd, Shanghai, China; ^3^ Shanghai University Mengchao Cancer Hospital, Shanghai, China; ^4^ Shanghai Cell Therapy Research Institute, Shanghai, China

**Keywords:** aging, aging symptoms, aging-related diseases, stem cell therapy, immune cell therapy

## Abstract

The extension of human lifespan has intensified the demand for developing more effective strategies to enhance quality of life. Age-related physiological decline and associated diseases now constitute significant societal challenges. As scientific understanding of aging mechanisms deepens, targeted intervention in the aging process is becoming increasingly feasible. Emerging evidence suggests that lifespan extension with preserved healthspan can be achieved through metabolic modulation and innovative molecular biology approaches. Notably, cell-based therapies demonstrate substantial anti-aging potential via multiple mechanisms including metabolic reprogramming, cellular repair systems, tissue regeneration, senescent cell clearance, and immunomodulation. This has catalyzed the emergence of cellular rejuvenation as a distinct discipline within anti-aging research. This review systematically examines current understanding of aging mechanisms, recent advancements in stem cell/immune cell technologies, and their clinical translation in age-related interventions. We further identify key challenges and future directions in the field, aiming to provide novel insights for extending human healthspan and improving geriatric care.

## 1 Introduction

Global life expectancy has demonstrated a remarkable upward trajectory since the establishment of the World Health Organization (WHO) in 1948. Initial records indicate an average global life expectancy of merely 46 years at the organization’s inception, whereas recent projections forecast this metric will reach 81 years in mainland China by 2035. This centennial progression reveals a consistent pattern of longevity gains, with human life expectancy increasing at an average rate of 2.5 years per decade (Vaupel et al., 2021). However, this demographic achievement coincides with critical biological constraints: post-quadragenarian physiology exhibits exponential declines in homeostatic recovery capacity, with advanced age correlating strongly with overt phenotypic manifestations of senescence (Pyrkov et al., 2021).

The WHO Global Report on Ageing and Health conceptualizes aging as a multidimensional biological phenomenon marked by the progressive accumulation of molecular and cellular damage. This deterioration fundamentally compromises stress response mechanisms and adaptive plasticity, ultimately culminating in disease pathogenesis or mortality. From an actuarial perspective, aging manifests operationally as a time-dependent exponential increase in mortality risk (Flatt and Partridge, 2018).

The seminal framework delineated by Kroemer et al. systematically classifies fourteen interconnected hallmarks of aging (Kroemer et al., 2025; Lopez-Otin et al., 2023). Genetic engineering interventions, particularly those validated in transgenic mammalian models, are increasingly recognized as promising therapeutic approaches for potential age reversal. These preclinical breakthroughs establish critical proof-of-concept for translating epigenetic reprogramming technologies into human rejuvenation strategies (Zhang et al., 2023). Beyer et al. conducted pioneering work elucidating evolutionarily conserved aging mechanisms through comparative analysis of RNA polymerase II transcriptional elongation kinetics (Debes et al., 2023). Contemporary research has substantially advanced our mechanistic understanding of aging pathophysiology through rigorous characterization of these biological hallmarks. This paradigm shift enables the development of precision anti-aging interventions targeting specific molecular pathways.

Current translational research in geroscience encompasses both preclinical investigations and early-phase clinical trials targeting fundamental aging mechanisms. Telomerase activation therapies attenuate telomeric attrition, thereby extending murine lifespan through telomere stabilization (Tomas-Loba et al., 2008). Rapamycin-mediated inhibition of the mTOR pathway enhances autophagic clearance, resulting in lifespan extension in *Saccharomyces cerevisiae* and murine models (Harrison et al., 2009; Walters and Cox, 2018). These interventions demonstrated good potential to treat age-related diseases and exhibited strong anti-aging effects (Gomes et al., 2013; Bonkowski and Sinclair, 2016). Anti-inflammaging interventions targeting chronic inflammation effectively mitigate multiple age-related comorbidities (Furman et al., 2019). The emerging field of cellular rejuvenation has gained substantial momentum following the seminal discovery of Yamanaka factors enabling epigenetic reprogramming of senescent cells (Takahashi and Yamanaka, 2006). Sinclair et al. demonstrated functional restoration of age-related vision loss in murine models through transient epigenetic remodeling (Lu et al., 2020).

The aging process in humans is ultimately attributable to cellular senescence (Massaro et al., 2023). The most fundamental anti-aging strategy necessitates targeted clearance of senescent cells, restoration of damaged cells, optimization of cellular metabolism, and maintenance of homeostatic balance. Immune cells serve pivotal functions in combating microbial infections and eliminating senescent cell populations. Stem cells not only modulate inflammatory responses and promote tissue regeneration, but also possess the capacity to differentiate into immune cells. Harnessing immunotherapeutic interventions and stem cell technologies constitutes a promising therapeutic strategy for decelerating aging processes (Baker et al., 2016; Gil and Withers, 2016). This review systematically examines the research progress regarding the role of immune cells and stem cells in the anti-aging process and provides scientific support for the new field of cellular anti-aging.

## 2 Anti-aging of stem cells

### 2.1 Relationship between stem cells and anti-aging

Stem cells constitute the biological foundation for tissue regeneration and repair mechanisms, while critically maintaining organismal metabolic homeostasis. The seminal importance of stem cell biology was recognized by the scientific community when *Science* designated breakthroughs in stem cell regeneration as one of the “Top 10 Scientific Breakthroughs of 1999”, highlighting their transformative potential in regenerative medicine (Bloom, 1999).

Adult stem cells serve as the cornerstone of endogenous regenerative capacity, orchestrating tissue homeostasis through dynamic self-renewal and repair mechanisms. Clinically significant depletion of these progenitor populations constitutes a hallmark of human aging pathogenesis (Nijnik et al., 2007). Age-related decline is also associated with a progressive impairment in stem cell differentiation capacity (Goodell and Rando, 2015). The aging process is characterized by the impairment of stem cell functionality and a compromised regenerative potential for regeneration. Preserving the integrity and function of stem cells is crucial for effectively mitigating the pathological manifestations of aging and the onset of age-related diseases (Chandel et al., 2016). Preclinical studies have revealed that age-related phenotypes can be delayed or reversed through xenotransplantation of young adipose mesenchymal stem cells into aged animals (Wang et al., 2023). Clinical trial data of stem cells against aging have demonstrated that intravenous injection of allogeneic mesenchymal stem cells can improve the levels of systemic health biomarkers and immunosenescence parameters of aging in frail patients (Liu et al., 2023a). However, the mechanisms by which stem cell senescence contributes to organismal aging remain to be fully elucidated.

### 2.2 Anti-aging mechanisms of stem cells

**FIGURE 1:**
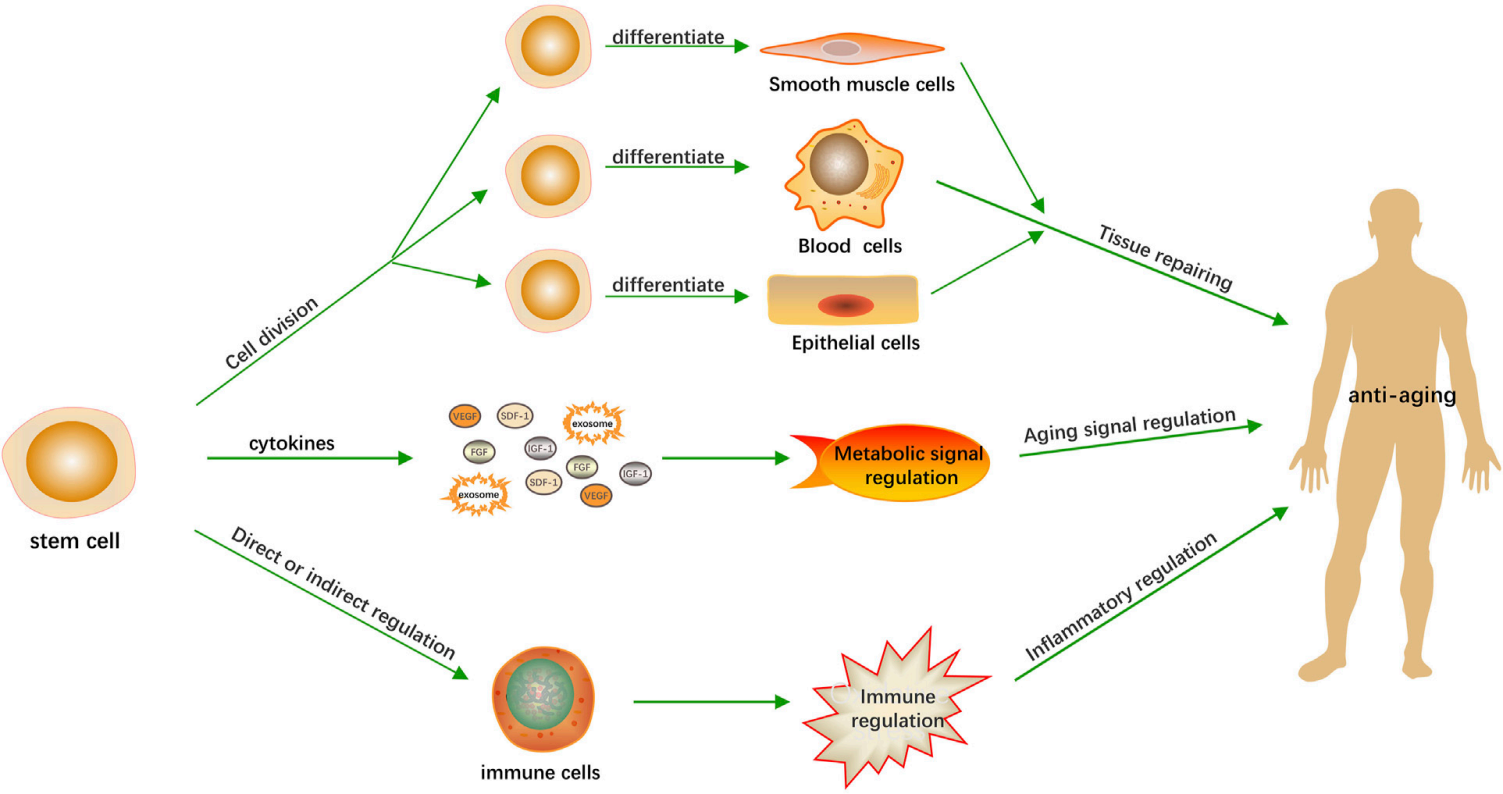
Anti-aging mechanisms of stem cells. Stem cells contribute to the anti-aging process through three primary mechanisms. Firstly, they facilitate tissue repair by undergoing division and differentiation to regenerate damaged or aged tissues. Secondly, they modulate the body’s metabolic activities by secreting growth factors that can influence cellular function and vitality. Thirdly, they assist in regulating the inflammatory response, which is crucial for maintaining immune system balance and preventing age-related inflammation.

Although the precise molecular mechanisms governing stem cell-mediated anti-aging effects require further characterization, emerging systems biology approaches provide conceptual frameworks for understanding their polytherapeutic nature. Stem cell therapies likely promote longevity through a multifaceted approach, encompassing tissue repair, metabolic regulation, and modulation of inflammatory processes (Figure 1). The mechanisms are not independent, but exhibit synergistic interactions.

The regenerative capacity of stem cells manifests through differentiation into functional cell lineages at injury sites. For instance, cardiac-engrafted stem cells demonstrate transdifferentiation potential into cardiomyocytes while stimulating *de novo* vasculogenesis (Bartolucci et al., 2017). Secondly, stem cells accelerate tissue repair and delay the tissue aging process by secreting growth factors, such as the tissue inhibitor of metalloproteinase-1 (TIMP-1) known to confer neuroprotective effects, vascular endothelial growth factor (VEGF) that promotes angiogenesis, fibroblast growth factor (FGF) that regulates cell proliferation and differentiation (Zhang et al., 2017). Exosomes, which contain anti-aging-related microRNAs (miRNAs), can influence aging-related signaling pathways, ultimately impacting the aging process (Ahmadi and Rezaie, 2021). Additionally, stem cells can enhance immune cell function through secreted factors or direct contact, leading to enhanced immune competence (Lee et al., 2012) and attenuated inflammatory responses (Cao et al., 2020). This multimodal therapeutic effect holds promise for treating age-related diseases with an immune component (Kuppa et al., 2022).

As organisms age, adult stem cells progressively lose their capacity to sustain tissue homeostasis and support regeneration. Senescent stem cells exhibit five hallmark characteristics: altered in depth of quiescence, changed in self-renewal propensity, altered cell fate, compromised stress resilience, and changed in population heterogeneity. Stem cell functionality is amenable to pharmacological and genetic editing-mediated regulation. Mammalian target of rapamycin (mTOR) inhibition via rapamycin rejuvenates regenerative competence in geriatric hematopoietic stem cells, whereas Yamanaka-mediated reprogramming potentiates visual system regeneration (Rando et al., 2025).

### 2.3 Preclinical research on stem cell anti-aging processes

Evidence from prior studies has indicated that stem cells from different sources have the potential to extend the lifespan of mice. For example, amniotic membrane-derived mesenchymal stem cells (AM-MSCs) and adipose tissue-derived mesenchymal stem cells (AD-MSCs) were intravenously transplanted into 10-month-old male F344 rats once a month throughout their lives. This treatment improved the cognitive and physical functions of naturally aging rats and prolonged their lifespan by 23.4% (AM-MSCs) and 31.3% (AD-MSCs), respectively (Kim et al., 2015). Similarly, muscular-derived stem cells (MD-SPCs) significantly increase the lifespan of prematurely aging mice (*Ercc1* ^*−/−*^ mice, *P* < 0.05) and aging-accelerated mutant mice (*Ercc1* ^*−/Δ*^ mice, aging score, *P* < 0.0008) (Lavasani et al., 2012). Stem cells harvested from young donors exhibit higher capacity to delay aging process. For instance, the injection of extracellular vesicles from conditioned media of bone marrow-derived mesenchymal stem cells (BM-MSCs) from young mice markedly increased the survival rate of *Ercc1* ^*−/−*^ mice (*P* = 0.005), reduced the percentage of SA-β-gal positive senescent BM-MSCs (*P* < 0.0001) and embryonic fibroblasts (MEFs, *P* = 0.0003), as well suppressed the levels of aging-related marker p16^INK4a^ (*P* = 0.0006) (Dorronsoro et al., 2021). Additionally, stem cells have proven effective in various animal disease models. Intravenous infusion of BM-MSCs prolonged the lifespan of spontaneously hypertensive rats by alleviating damage to multiple end-organ systems (survival rate and average lifespan: control group, 30.7%, 176.1 ± 1.8 days, MSC group, 70.6%, 183 ± 2.4 days)and activating the TGF-β-SMAD3/FOXO1 pathway in different tissues (kidneys, brains, hearts and liver) (Nakazaki et al., 2020). Collectively, these results indicate that stem cells can delay or reverse the decline in physiological functions associated with aging and the onset of age-related diseases, extending both healthspan and overall lifespan.

### 2.4 Clinical research in stem cell anti-aging

#### 2.4.1 Clinical research on stem cells improving aging symptoms

A review of 11 clinical trials retrieved from a clinical trial database (Supplementary Table S1) investigating stem cell therapies for anti-aging symptoms revealed that MSCs were the predominant cell type used (81.8%, 9 out of 11 trials). Notably, allogeneic BM-MSCs (a cellular therapy called Lomecel-B) developed by Longeveron company has shown promising initial clinical efficacy in treating aging-related frailty (see Table 1 for details). In a Phase I clinical trial (NCT02065245) (Golpanian et al., 2016; Golpanian et al., 2017) 15 elderly subjects (mean age: 78.4 years, with a frailty score ranging from four to seven on the Clinical Frailty Scale) received intravenous Lomecel-B. Six months after administration, the treatment group showed improved 6-min walk distance (6MWT), accompanied by reduced levels of the inflammatory marker TNF-α. Moreover, a dose of 1 × 10^8^ MSCs led to a 76.6-m increase in walking distance after 6 months, which could be regarded as a “substantial change” (improvement of more than 49 m) (Perera et al., 2006). Significant improvements were also observed in forced expiratory volume (FEV1), cognitive status (MMSE), and SF-36 questionnaire scores. Following the promising Phase I results, a Phase II randomized, double-blind, placebo-controlled trial (Tompkins et al., 2017) was conducted, in which the elderly subjects in the 1 × 10^8^ group exhibited better outcomes in quality of life and functional status. None of the 30 participants experienced any treatment-emergent serious adverse events (TE-SAEs), consistent with the Phase I findings. Importantly, the expression of CD8 T cell markers was significantly reduced in the 2 × 10^8^ group; the expansion of these cells is known to occur with aging (McElhaney and Effros, 2009), suggesting that stem cells may extend the healthy lifespan of frail elderly individuals. The Phase IIb clinical trial (NCT03169231) was conducted to assess the population’s dose response to Lomecel-B (Yousefi et al., 2022). By day 180, the 6MWT in the higher-dose groups (5 × 10^7^, 1 × 10^8^, 2 × 10^8^) had increased significantly, and a significant dose-response relationship was observed. Further clinical investigations on Lomecel-B are ongoing (jRCT2043200038 and NCT02982915). These trials position Lomecel-B as a potential candidate for advancing MSC therapy for frailty to Phase III clinical trials-the final stage before regulatory approval.

**TABLE 1:** Key outcomes from clinical trials in frail elderly patients.

Study (year) symptom	Intervention (Dose)	Primary endpoint	Efficacy outcome (Result; 95% CI; *P*)	Evidence level	Limitations
NCT02065245 (2017) Aging Frailty	Allogeneic BM-MSCs^a^ 2 × 10^7^ cells 1 × 10^8^ cells 2 × 10^8^ cells	TE-SAEs^b^ (1 month postinfusion)	No TE-SAEs^b^ vs. baseline 6MWD^c△^ (3 months, *P* = 0.02; 6 months, *P* = 0.001) Serum TNF-α^d^^▽^(6 months, *P* < 0.0001) SF-36^e△^ Physical Component Score (1 × 10^8^, *P* < 0.05)	Nonrandomized, dose-escalation study	Small sample size all white race
NCT02065245 (2017) Aging Frailty	Allogeneic BM-MSCs^a^ Placebo 1 × 10^8^ cells 2 × 10^8^ cells	TE-SAEs^b^ (1 month postinfusion)	No TE-SAEs^b^ vs. baseline, 6 months 6MWD^c△^, SPPB^f^^△^, FEV1^g^^△^, Serum TNF-α^d^^▽^, SQOL-F^h△^, (1 × 10^8^,*P* < 0.05) % CD8 T-cells^▽^ (2 × 10^8^, *P* = 0.022) %B cells expressing intracellular TNF-α^d▽^ (1 × 10^8^, *P <* 0.0001; 2 × 10^8^, *P* = 0.002)	Randomized, double-blinded, dose-finding study	Small sample size The reasons underlying the inverse dose relationship remain incompletely understood
NCT03169231 (2022) Aging Frailty	Allogeneic BM-MSCs^a^ Placebo 2.5 × 10^7^ cells 5 × 10^7^ cells 1 × 10^8^ cells 2 × 10^8^ cells	Change from baseline in 6MWT^c^ compared to placebo (6 months postinfusion)	6MWD^c△^ (5 × 10^7^, 27.7, −8.1–63.5) 6MWD^c△^ (1 × 10^8^, 16.8, −18.5–52.2) 6MWD^c△^ (2 × 10^8^, 41.3, −2.4–84.9) significant dose-response relationship	Multicenter, randomized, parallel-arm, double-blinded And placebo-controlled phase 2b trial	Not found
ChiCTR-OOh-17011878 (2022) Aging Frailty	Autologous NK cells Not found dose	Changes in T-cell senescence and exhaustion, as well as SASP (1 and 4 weeks postinfusion)	vs. baseline, 1 and 4 weeks Senescent CD4^+^T cells (CD4^+^CD28^−^, CD4^+^CD57^+^, CD4^+^KLRG1^+^, CD4^+^CD28^−^ CD57^+^, CD4^+^CD28^−^KLRG1^+^, *P* < 0.01) Senescent CD8^+^T cells, *P* < 0.05 exhausted T cells (CD4^+^PD-1^+^, CD8^+^PD-1^+^, CD4^+^TIM-3^+^, CD8^+^TIM-3^+^, *P* < 0.05) SASP related factors (IL-6^i^, IL-8, IL-1a, IL-17, MIP-1a, MIP-1b, and MMP1, *P* < 0.001)	Not found	Lack of pathological assessment

^△^increased; _▽_decreased; ^a^bone marrow-derived mesenchymal stem cells; ^b^treatment-emergent serious adverse events; ^c^6-minute walk distance; ^d^tumor necrosis factor; ^e^36-Item Short Form Health Survey; ^f^Short physical performance battery; ^g^Forced expiratory volume after 1 s; ^h^Sexual Quality of Life-Female.C; ^i^interleukin.

#### 2.4.2 Clinical research on the application of stem cells in skin rejuvenation

Skin rejuvenation is a major focus in stem cell-based anti-aging research, and numerous clinical trials are underway to evaluate interventions for enhancing skin rejuvenation (Supplementary Table S2). Ichihashi et al. injected autologous adipose-derived stem cells (ADSCs) into the skin of eight participants with signs of skin aging (Ichihashi et al., 2023). By analyzing patient photographs before injection and at 1, 3, 6, and 12 months post-injection, They found that the average improvement rates—calculated as [(1-A/B) × 100%, where A and B represent the respective 5-grade visual scale scores after and before the treatment, respectively] —were 33.3%–40% for facial wrinkles, nasolabial fold depth, and lower eyelid drooping. These findings suggest that a single ADSCs injection may restore skin health and a youthful appearance, offering a promising non-surgical rejuvenation approach. In another study, Liang et al. investigated the synergistic effect of microneedling (MN) and human umbilical cord-derived mesenchymal stem cells conditioned media (hUC-MSCs-CM) on skin aging (ChiCTR-INR-17013311). The MN+hUC-MSCs-CM group demonstrated significantly greater improvements in skin brightness and texture compared to MN alone (*P* < 0.05) (Liang et al., 2022). Yusharyahya et al. compared two different delivery methods for AD-MSCs secretome in skin rejuvenation (NCT05508191). They found that both MN and fractional carbon dioxide lasers (FL) delivery methods significantly enhanced the total Dermoscopy Photoaging Scale (DPAS) (*P* < 0.01) and reduced wrinkles (*P* < 0.001), indicating that these methods could optimize skin rejuvenation (Yusharyahya et al., 2023). (see Table 2 for details)

**TABLE 2:** Key outcomes from clinical trials in skin rejuvenation.

Study (Year) Symptom	Intervention (Dose)	Primary endpoint	Efficacy outcome (Result; 95% CI; *P*)	Evidence level	Limitations
Case Report^a^ (2023) skin rejuvenation	Autologous ADSCs^b^ 1 × 10^8^ cells	Skin manifestations (before and 1, 3, 6, and 12 months after injections)	vs. Baseline Facial wrinkles, nasolabial fold depth, and lower eyelid drooping^▽^ (average improvement rates: 33.3%–40%)	Case Report (8 patients)	Only used photo comparisons
ChiCTR-INR-17013311 (2022) skin rejuvenation	Allogeneic hUC-MSCs-CM^c^ MN alone (MN saline) 2.0 mL hUC-MSCs-CM^c^ +MN^d^	Clinical improvement of both sides of the face (3 months paint+MN^d^)	vs. MN alone, 3 months Melanin Index^▽^, Brown Spots^▽^, *P* = 0.00 Ultraviolet Spots^▽^, *P* < 0.05 Wrinkles^▽^, Pores^▽^, *P* = 0.00 Elasticity^△^, *P* = 0.00	Randomized controlled split-face study	Small sample size Lack of pathological assessment
NCT05508191 (2023) skin rejuvenation	Allogeneic AD-MSCs^e^ secretory body MN^d^ FL^f^	Clinical improvement of two methods of delivery (6 weeks after delivery)	vs. baseline DPAS^g▽^ (MN^d^, *P* < 0.01; FL^f^, *P* < 0.01) Wrinkles^▽^,ebum^▽^, porphyrin^▽^, (MN^d^, *P* < 0.001; FL^f^, *P* < 0.001) MN^d^ vs FL^f^, no significant differences	Single-blind, randomized split-face clinical trial	Relatively short duration (6 weeks) of trial serves

^△^increased; _▽_decreased; ^a^A Single Intradermal Injection of Autologous Adipose-Tissue-Derived Stem Cells Rejuvenates Aged Skin and Sharpens Double Eyelids; ^b^adipose-derived stem cells; ^c^human umbilical cord mesenchymal stem cell conditioned culture medium; ^d^microneedling; ^e^adipose tissue-derived mesenchymal stem cells; ^f^fractional CO_2_ laser; ^g^dermoscopy photoaging scale.

#### 2.4.3 Progress of clinical research in stem cells in age-related diseases

Currently, several age-related diseases remain incurable, such as neurodegenerative disorders, cardiovascular and cerebrovascular diseases, and autoimmune diseases (Liu et al., 2023b). Prolonged drug administration often lead to unintended side effects. In contrast, stem cell therapy offers a promising alternative that avoids these adverse effects while effectively treating age-related diseases (see Tables 3, 4 for details).

**TABLE 3:** Key outcomes from clinical trials in neurodegenerative diseases.

Study (Year) Symptom	Intervention (Dose)	Primary endpoint	Efficacy outcome (Result; 95% CI; *P*)	Evidence level	Limitations
NCT02600130 (2022) AD^a^	Allogeneic BM-MSCs^b^ Placebo 2 × 10^7^ cells 1 × 10^8^ cells	TE-SAEs^c^ (26 weeks postinfusion)	vs. Placebo VEGF^d△^ (2 × 10^7^, *P* <0.05; 1 × 10^8^, *P* < 0.001) IL-4^e△^ (2 × 10^7^, *P* <0.001; 1 × 10^8^, *P* < 0.05) IL-6^e△^ (1 × 10^8^, *P* <0.001) D-Dimer^△^, sIL-2Rα^f△^, left hippocampus^△^ (1 × 10^8^, *P* < 0.05) IL-10^e△^, MMSE^g△^ (2 × 10^7^, *P* <0.05) IL-12^e△^ (2 × 10^7^, *P* <0.001)	Double-blind, randomized trial	The efficacy assessment was inadequately conducted
NCT02611167 (2021) PD^h^	Allogeneic BM-MSCs^b^ 1 × 10^6^ cells 3 × 10^6^ cells 6 × 10^6^ cells 10 × 10^6^ cells	Transfusion reaction Study-related adverse events, and immunogenic Responses (12 months postinfusion)	No TE-SAEs^c^ vs. baseline, 10 × 10^6^ cells TNF-α^h▽^, CCL22^i▽^, BDNF^j△^, *P*< 0.05 Pseudo-continuous arterial spin (time, region, perfusion^△^, *P* < 0.001) UPDRS^k▽^ (motor scores, *P* < 0.01, total scores, *P* < 0.05)	Single-center open label dose-escalation trial	Small sample size Lack of genetic testing subtyping The lack of a placebo group
NCT01297413 (2019) stroke	A alloischemic tolerance BM-MSCs^b^ Phase 1: 0.5,1,1.5 × 10^8^ cells Phase 2: 1.5 × 10^8^ cells	No TE-SAEs^c^ (12 months postinfusion)	No TE-SAEs^c^ vs. baseline Barthel Index scores (6, 12 months, *P* < 0.001) patient proportion^△^ (Barthel score ≥ 95, baseline, 11.4%; 12 months, 35.5%)	Multi-center, open-label, randomized study	No control group Mechanism of action was not studied

^△^increased; _▽_decreased; ^a^alzheimer’s disease; ^b^bone marrow-derived mesenchymal stem cells; ^c^treatment-emergent serious adverse events; ^d^vascular endothelial cell growth factor; ^e^interleukin; ^f^Soluble IL-2 receptor α; ^g^Mini–Mental State Examination; ^h^tumor necrosis factor; ^i^chemokine ligand; ^j^brain-derived neurotrophic factor; ^k^Unified Parkinson’s Disease Rating Scale.

**TABLE 4:** Key outcomes from clinical trials in cardiovascular diseases and autoimmune diseases.

Study (Year) Symptom	Intervention (Dose)	Primary endpoint	Efficacy outcome (Result; 95% CI; *P*)	Evidence level	Limitations
NCT01458405 (2020/2021) AMI^a^	Allogeneic cardiac stem cells (CDCs) Placebo 2.5 × 10^7^ cells	TE-SAEs^b^ (1 month intracoronary injection) infarct size (12 months intracoronary injection)	No TE-SAEs^b^ vs. Placebo, 6 months Scar size, no significant differences NT-proBNP^c▽^, *P* < 0.05 vs baseline, 6 months LVEDV^d▽^, LVESV^e▽^, *P* < 0.05 Segmental Ecc^f^ (segments containing scar tissue, *P* < 0.05)	Multicentre, randomized, double-blinded, placebo-controlled trial	The trial was interrupted before completion of the 12-month follow-up A single administration Stop-flow intracoronary delivery
NCT01739777 (2017) heart failure	Allogeneic UC-MSCs^g^ Placebo 1 × 10^6^ cells/kg	TE-SAEs^b^, clinical improvement (12 months postinfusion)	No TE-SAEs^b^ vs. baseline, 3, 6, 12 months LVEF^h^ (echocardiographic parameters, *P* < 0.01; CMR^i^ measurements, 6 months, *P* < 0.01) NYHA^j^ (12 months, *P* < 0.01) MLHFQ^k^, VE/VCO2^l^ (12 months, *P* < 0.05)	A phase 1/2, randomized, double-blind, placebo-controlled clinical trial	Small sample size No measurement of myocardial perfusion and fibrosis
NCT01547091 (2019) RA^m^	Allogeneic WJ-MSCs^n^ 2 × 10^7^ cells	Safety (serological markers tests, 1 year and 3 years postinfusion) Efficacy (DAS28^o^ and HAQ^p^, 1 year and 3 years postinfusion)	vs. baseline, 1 year and 3 years No abnormality in the blood routine examination ESR^q▽^, CRP^r▽^, *P* < 0.001 RF^s▽^(3 years, *P* < 0.05) DAS28^o^ score, HAQ^p^ score, *P* < 0.001	Not found	All patients from a single center No placebo control for the long-term observation

_▽_decreased; ^a^acute myocardial infarction; ^b^treatment-emergent serious adverse events; ^c^N-terminal pro b-type natriuretic peptide; ^d^ventricular end-diastolic volume; ^e^end-systolic volume; ^f^circumferential strain; ^g^umbilical cord mesenchymal stem cells; ^h^left ventricular ejection fraction; ^i^cardiac magnetic resonance; ^j^New York Heart Association; ^k^Minnesota Living with Heart Failure Questionnaire; ^l^ventilatory efficiency; ^m^rheumatoid arthritis; ^n^umbilical cord-derived Wharton’s jelly MSCs; ^o^28-joint disease activity score; ^p^Health Assessment Questionnaire; ^q^erythrocyte sedimentation rate; ^r^C-reaction protein; ^s^Rheumatoid Factor.

In a clinical trial of Alzheimer’s disease (AD) (NCT02600130), researchers observed that compared to the placebo group, a single intravenous infusion of Lomecel-B reduced interleukin levels (IL-4 and IL-6), significantly increased vascular endothelial cell growth factor (VEGF), attenuated inflammatory response, and improved vascular function (Brody et al., 2023). These phase I trial findings support the hypothesis that neuroinflammation and vascular impairment are key pathophysiological mechanisms in AD. Based on these results, a subsequent phase IIa trial (NCT05233774) is evaluating potential therapeutic effects on cognitive function and AD biomarkers in participants receiving single or multiple doses of Lomecel-B. Another Parkinson’s disease (PD) trial (NCT02611167) demonstrated that a single intravenous infusion of allogeneic BM-MSCs significantly reduced peripheral inflammation (*P* < 0.05), increased brain-derived neurotrophic factor levels (*P* < 0.05), and improved the OFF status (OFF:12-h medication withdrawal before assessment) in all patients (Schiess et al., 2021). At 52 weeks, clinically meaningful reductions in PDQ-39 scores were observed across the cohort. This study represents the first clinical evidence that a single intravenous administration of allogeneic HMSCs can ameliorate PD symptoms. In a chronic stroke trial (NCT01297413), intravenous infusion of allogeneic ischemic-tolerant mesenchymal stem cells increased Barthel Index score by 10.8 ± 15.5 points (*P* < 0.001) at 12 months post-treatment. The proportion of patients achieving good functional results (Barthel score ≥ 95) rose from 11.4% at baseline to 35.5% at 12 months (Levy et al., 2019). Behavioral endpoints also showed universal improvement, indicating functional recovery. To date, this remains the largest clinical trial of intravenous mesenchymal stem cells therapy in chronic stroke patients, with no serious adverse events reported. Additional clinical trials investigating stem cells therapies for neurodegenerative disorders have yielded promising results (Supplementary Table S3).

A 2020 phase I/II study (NCT01458405) evaluated the therapeutic effects of cardiosphere-derived cells (CDCs) in patients with acute myocardial infarction (AMI). The study demonstrated that CDCs administration achieved positive secondary efficacy endpoints, mitigated left ventricular remodeling and normalized cardiac biomarker levels. Compared with the placebo group, CDCs-treated patients exhibited significantly lower ventricular end-diastolic volume (LVEDV) and end-systolic volume (LVESV) at 6 months (*P* = 0.02), along with reduced N-terminal pro b-type natriuretic peptide (NT-proBNP) levels (*P* = 0.02) (Makkar et al., 2020). Subsequent analyses revealed that CDC therapy enhanced myocardial segmental function, particularly in scarred segments, with concomitant improvements in circumferential strain (Ecc) (Ostovaneh et al., 2021). The NCT01739777 clinical trial demonstrated that intravenous infusion of umbilical cord mesenchymal stem cells (UC-MSCs) was safe and reduced ejection fraction in patients with stable heart failure (Bartolucci et al., 2017). The treatment group showed consistent improvements in left ventricular ejection fraction (LVEF) at all follow-up intervals (3, 6, and 12 months). Furthermore, these patients exhibited clinically meaningful enhancements in functional capacity (as measured by the New York Heart Association functional classification) and quality of life (assessed by the Minnesota Living with Heart Failure questionnaire score) (p-value). These findings indicate that the intervention may improve both cardiac function and patient wellbeing. Currently, several large-scale multicenter trials are underway (Supplementary Table S4).

A notable advantage of stem cells therapy is its favorable safety profile and high efficacy in treating autoimmune diseases (Supplementary Table S5). A 2019 prospective phase I/II study conducted in China (NCT01547091) evaluated the long-term effects of intravenous infusion of UC-MSCs combined with low-dose DMARDs (disease-modifying anti-rheumatic drugs) in patients with rheumatoid arthritis (RA) (Wang et al., 2019). The study demonstrated that UC-MSCs treatment produced stable and beneficial effects lasting up to 3 years, evidenced by significant improvements in patients’ quality of life and partial reversal of joint deformities in some cases. Additionally, routine blood tests, liver and kidney function, and immunoglobulin levels at 1 and 3 years post-treatment showed no abnormalities, while inflammation markers (ESR, CRP, RF, and anti-CCP) significantly decreased compared to baseline (*P* < 0.05). Further analysis revealed significant reductions in Health Assessment Questionnaires (HAQ) and 28-Joint Disease Activity Score (DAS28) after treatment (*P* < 0.05) (Wang et al., 2019).

## 3 Anti-aging of immune cells

### 3.1 The relationship between immune cells and anti-aging

The aging process involves a decline in the immune system’s ability to respond to pathogens and cancer cells, indicating that the immune system itself ages (Zhou et al., 2021). Studying the aging of different immune cells could uncover the mechanisms behind this immune aging throughout life.

Thymus degeneration contributes to immune aging. As age increases, the number of naive CD4^+^ and CD8^+^ T cells decreases, while the number of memory and effector T cells increase (Qin et al., 2016). The decline in T cells with age is evident in several ways: reduced responsiveness to specific cytokines, decreased cytotoxic activity of CD8^+^ T cells, and a increased loss of CD28 receptor on T cells. Notably, senescent T cells, often identified by the CD45RA^+^CD8^+^ T cell phenotype, exhibit a senescence-associated secretory phenotype (SASP). This SASP is characterized by increased production of pro-inflammatory cytokines such as IL-18 and ADAM28, contributing to a chronic inflammatory state (Li et al., 2023).

NK cells and cytotoxic CD8^+^ T cells co-regulate the task of immune surveillance and the clearance of stressed cells. Compelling evidence indicates that aging affects the diversity of NK cell subsets, thereby altering the functional features of human NK cells. Specifically, the number of CD56^bright^ NK cells decreases with age (Jiao et al., 2009; Camous et al., 2012; Salam et al., 2013; Brauning et al., 2022), and similarly, the secretion of certain cytokines and chemokines by NK cell subsets, such as IFN-γ, IL-2, IL-12, MIP-1α, and IL-8, also declines. These changes are consistent with a decline in NK cell function and may represent key features of immunosenescence and inflammaging. Additionally, the percentage of CD56^dim^CD57^pos^ NK cells increases with age (Salminen, 2021). In older individuals, the expression of natural cytotoxicity triggering receptor 1 (NCR1 or NKp46) and 3 (NCR3 or NKp30) in CD56^bright^ and CD56^dim^ NK cell subsets significantly decreases, which impairs the NK cell immune surveillance functions. Furthermore, with ageing, the release of perforin in human NK cells and its binding to target cells at the immune synapse are significantly reduced, thereby inhibiting NK cell cytolysis activity.

A study reveals the profound impact of immune system aging on the deterioration of solid organs (Yousefzadeh et al., 2021). Researchers engineered a mouse model to simulate the senescent immune cells by selectively deleting critical genes within hematopoietic stem cells. The genetically modified mice exhibited accelerated immune senescence after adolescence, marked by a decline in immune cells numbers, reduced spleen and thymus sizes, increased expression of cellular senescence markers, and elevated levels of senescent-related biomarkers with cellular aging in various tissues. Notably, transplanting senescent splenocytes from old mice into young ones accelerated the recipients’ aging. Conversely, infusing splenocytes from young mice into old mice with impaired immune systems slowed their aging. These results emphasize that senescent immune cells can promote systemic aging, whereas youthful immune cells can mitigate the aging process.

### 3.2 Anti-aging mechanisms of immune cells

Immune cells contribute to anti-aging by modulating the body’s inflammatory response. Specifically, studies have shown that NK cells directly kill senescent cells through the NKG2D receptor and granule exocytosis mechanism (Sagiv et al., 2016; Antonangeli et al., 2019). NK cells recognize and eliminate senescent cells through a complex interplay of receptors and ligands on their surfaces. Among these, the NKG2D receptor plays a critical role by recognizing a variety of ligands expressed by stressed cells, including MICA, MICB, and members of the UL16 binding protein (ULBP) family. Senescent cells upregulate these ligands in response to stress or damage, which activates NK cells (Sagiv et al., 2016). Additionally, senescent or damaged cells express lower levels of MHC I molecules, resulting in reduced inhibition of NK cells and consequently enhanced NK cell-mediated killing (Bryceson and Long, 2008).

**FIGURE 2:**
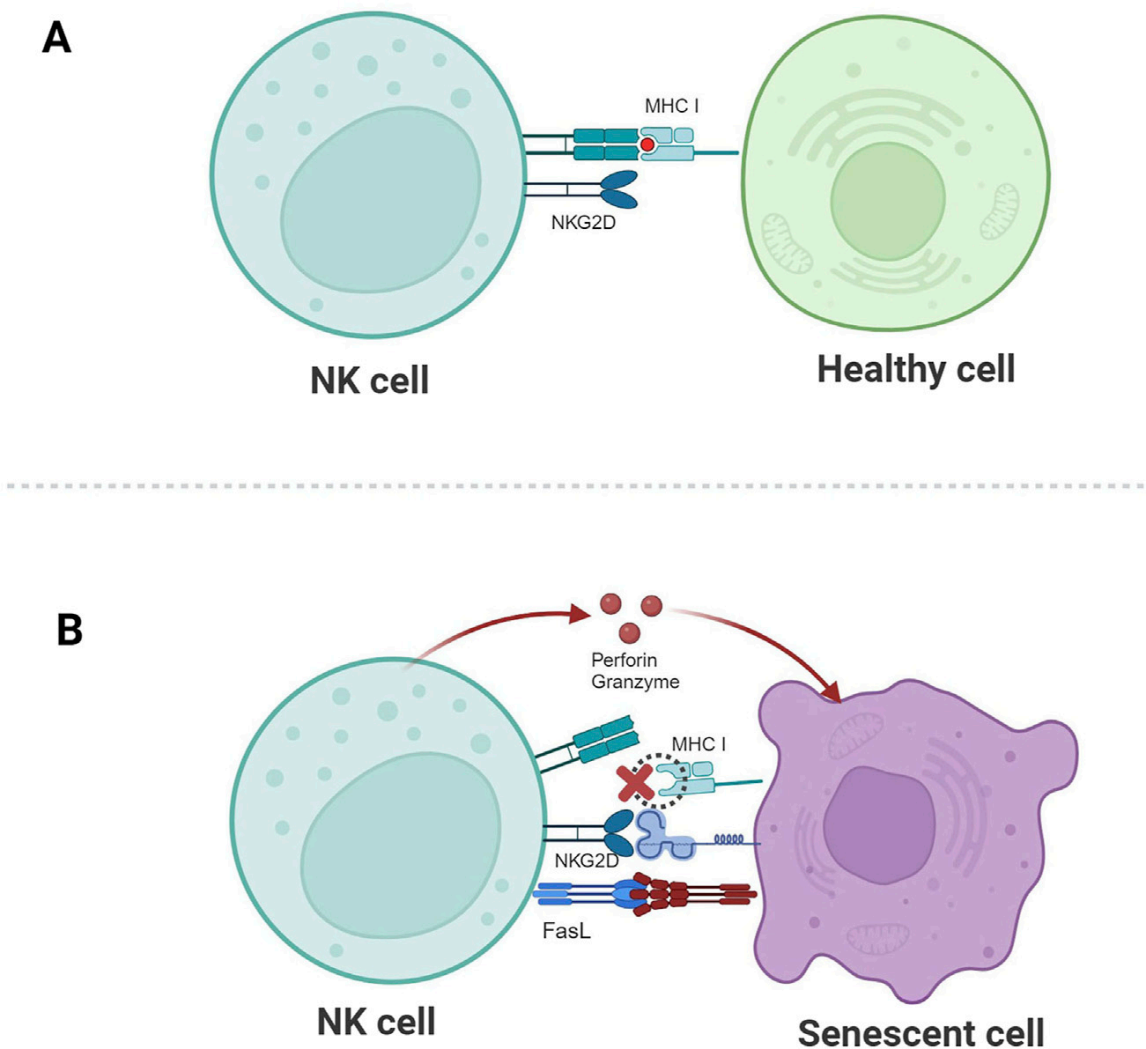
Schematic illustration of NK cell-mediated recognition and clearance of senescent cells. **(A)** The corresponding ligands for NKG2D are not expressed, and the inhibitory signal mediated by MHC I molecules plays a dominant role, so healthy cells are not killed. **(B)** The expression of MHC I molecules is downregulated, the activating signal mediated by NKG2D plays a dominant role, and NK cells kill senescent cells through granule exocytosis or by connecting death receptor-induced apoptosis.

The cytotoxic effects of NK cells are mediated through two main mechanisms: granule exocytosis and death receptor ligation (Figure 2) (Prata et al., 2018). In the death receptor pathway, Fas ligand (FASL) or TNF-related apoptotic inducing ligand (TRAIL) on NK cells bind to their respective receptors (Fas or TRAIL-R) on senescent cells, activating the extrinsic apoptosis pathway and leading to target cell death (Hazeldine and Lord, 2013). In the granule exocytosis pathway, NK cells release perforin and granzymes upon recognizing target cells. Perforin forms pores on the target cell membrane, allowing granzymes to enter and induce apoptosis (Sagiv et al., 2013).

Similarly, genetically modified T cells, such as CAR-T cells, can be engineered to target senescent cells. When CAR-T cells bind to their target cells via the chimeric antigen receptor, they receive activation signals that lead to the formation of non-classical immune synapses, thereby activating the CAR-T cells (Li et al., 2020; Cassioli et al., 2022). Once activated, CAR-T cells kill target cells through three pathways: the perforin/granzyme pathway, which is directly triggered by the CAR recognition; and the death receptor pathway and cytokine pathway, which may not require direct CAR-antigen interaction (Lemoine et al., 2021). Notably, the mechanisms by which T cells and NK cells kill senescent target cells are similar.

### 3.3 Preclinical research on the anti-aging of immune cells

The development of CAR-T therapy has provided researchers with a new approach for developing anti-aging agents. By engineering CAR-T cells to recognize specific markers on senescent cells, anticancer therapies can be adapted for anti-aging purposes. For instance, Amor et al. identified urokinase-type plasminogen activator receptor (uPAR) as a unique surface marker on senescent cells through systematic screenings (Amor et al., 2020). CAR-T cells designed to target uPAR can effectively eliminate senescent cells in mouse models, leading to improvements in metabolic function and tissue homeostasis. However, while these murine studies show promise, the translation to human applications requires further validation, as current research is still in exploratory stages.

Leveraging the high expression of NKG2D ligands on senescent cells, Yang et al. developed NKG2D-CAR-T cells (Yang et al., 2023). *In vitro* assays demonstrated that these cells can specifically target human senescent cells induced by various stressors, carcinogenic stress, replicative exhaustion, DNA damage, or p16^INK4a^ overexpression. Furthermore, the levels of cytokines (TNF-α and IFN-γ) and effector molecules (granzyme B and perforin) produced by these CAR-T cells significantly increased (*P* < 0.01). *In vivo*, injection of mouse NKG2D-CAR-T cells into radiation-induced senescent mice and naturally aged 24-month-old mice (equivalent to 80-year-old humans) resulted in significant improvements in physical function such as bones health, muscles strength, motor abilities. Additionally, senescence-associated markers (Rae-1, Mult-1, H60b) were reduced, and treated natural aged mice exhibited better skeletal and motor functions at 30 months of age. Extending these findings to non-human primates, the researchers administered autologous NKG2D-CAR-T cells into Rhesus and crab-eating macaques, whose ages corresponded to 60-year-old humans. Two months post-treatment, a significant reduction in SA-β-gal-positive cells in adipose tissue was observed (*P* < 0.05). Importantly, blood chemistry analyses, including ALT and AST levels, remained unchanged compared to pre-treatment values, indicating the safety of this approach. These results suggest that NKG2D-CAR-T cells could serve as a safe and effective anti-aging therapy.

In a different context, Earls et al. discovered NK cells in the brains of patients with synucleinopathy and in adult mice (Earls et al., 2020). Further investigations revealed that human NK cells can effectively clear extracellular alpha-synuclein aggregates via pathways involving TLR4 and TLR2. Interestingly, while extracellular α-synuclein (α-syn) aggregates do not overly stimulate NK cells effector functions, they do reduce the production of IFN-γ and may modulate NK cell cytotoxicity. In a preclinical mouse model of Parkinson’s disease (PD), depletion of NK cells exacerbated motor impairments and increased the accumulation of phosphorylated α-syn. These observations suggest that NK cells play a protective role by clearing α-syn aggregates and possibly through IFN-γ production, thereby potentially delaying or mitigating the progression of PD and other synuclein-related neurodegenerative disorders.

### 3.4 Clinical research on the anti-aging of immune cells

#### 3.4.1 Clinical research on immune cells in the aspect of aging symptoms

Aging is often accompanied by a decline in immune system function, particularly in T cell immune responses. A recent clinical trial in China (ChiCTR-OOh-17011878; Supplementary Table S1; see Table 1 for details) showed that, compared with the control group (n = 5), intravenous injection of NK cells (n = 32) reduced the number of senescent T cells in patients (such as PD-1^+^, TIM-3^+^ T cells). Additionally, SASP-related factors—including IL-6, IL-8, IL-1α, IL-17, MIP-1α, and MMP1—were significantly decreased. The results indicated that autologous NK cell therapy mitigated cellular senescence (Tang et al., 2022). Another clinical trial demonstrated that infusing *in vitro*-expanded and activated autologous NK cells reduced the expression of senescence markers (p16 and β-galactosidase) in peripheral blood mononuclear cells (PBMCs), as well as levels of inflammatory markers such as ferritin, monocyte chemoattractant protein-1 (MCP-1), IL-6, and IFN-γ. The study found that one or two NK cell infusions were safe, alleviated aging-related features (including inflammation and immunosenescence), and improved overall health (Chelyapov et al., 2022).

#### 3.4.2 Clinical research on immune cells in the aspect of aging diseases

A phase I clinical trial (NCT04678453) is currently underway to evaluate the safety, tolerance, and efficacy of autologous NK cell therapy (SNK01) as a monotherapy for AD patients. Preliminary results indicate that SNK01 is well tolerated, with no serious adverse events observed, and no dose-limiting toxicity reached. At the 11-week evaluation, most patients exhibited stable or improved scores on multiple cognitive function assessments, including CDR-SB, ADAS-Cog, and MMSE. One patient receiving the highest dose (4 × 10^9^ cells) demonstrated significant improvement in all three aforementioned scores. Additionally, cerebrospinal fluid biomarker levels remained stable or showed improvement. These findings provide preliminary evidence supporting the potential use of NK cells in AD treatment.

In 2022, German scientists intravenously administered autologous CD19 CAR-T cell therapy to five patients with systemic lupus erythematosus (SLE) and demonstrated its tolerability and efficacy (Mackensen et al., 2022). By 3 months post-treatment, all patients met the criteria for SLE remission, with the SLE Disease Activity Index (SLEDAI-2K) decreasing significantly to nearly zero. The therapy markedly reduced B-cell levels, correlating with improvements in clinical symptoms and laboratory parameters—including seroconversion of anti-double-stranded DNA (anti-dsDNA) antibodies to negative. The treatment was well tolerated, with only mild cytokine release syndrome (CRS) reported. In 2023, the same research team successfully applied CD19 CAR-T cell therapy to a patient with anti-synthetase syndrome (ASS). The patient exhibited significant improvements in muscle strength and endurance, along with marked reductions in creatine kinase and myoglobin levels. Notably, anti-Jo-1 antibody titers declined from 331 to 5 U/L. Additionally, respiratory symptoms improved, and chest CT scans confirmed resolution of lung inflammation. Even after discontinuation of all immunosuppressive medications, the patient’s condition continued to improve (Muller et al., 2023). These finding represent a major breakthrough in autoimmune disease treatment, marking CAR-T therapy’s successful expansion to a second autoimmune disorder after SLE.

## 4 Future outlook

In recent years, significant progress has been made in the application of cell therapy for anti-aging, with numerous clinical trials currently underway (Hickson et al., 2019; Justice et al., 2019; Nambiar et al., 2023). Nevertheless, risks persist. When using iPSCs for diabetes treatment, residual undifferentiated cells may form teratomas. A case study reported that a type 2 diabetes patient developed a mass at the injection site 2 months after receiving iPSC-derived pancreatic β-cell therapy. Histological examination revealed positive staining for OCT3/4 and SOX2 in the mass (Han et al., 2022), confirming its pluripotent stem cell origin (Rizzino and Wuebben, 2016). Although mesenchymal stem cells (MSCs) are generally regarded as safe, non-standardized culture conditions and administration protocols may potentially lead to tumorigenic complications. This was illustrated in a case report involving a 66-year-old male patient who developed glial proliferative lesions following intrathecal administration of MSCs obtained from an unverified source (Berkowitz et al., 2016). CAR-T cell therapy is frequently complicated by three principal toxicities: cytokine release syndrome (CRS), immune effector cell-associated neurotoxicity syndrome (ICANS), and immune effector cell-associated hemophagocytic lymphohistiocytosis (HLH)-like syndrome (IEC-HS). These treatment-related complications uniformly manifest through cytokine storm-mediated systemic hyperinflammation (Hughes et al., 2024).

The potential risks associated with these therapies can be effectively mitigated through three primary approaches: (1) implementing highly sensitive detection methods to monitor and minimize residual undifferentiated pluripotent stem cells; (2) adhering to standardized manufacturing protocols and stringent quality control measures; and (3) utilizing targeted interventions such as tocilizumab for cytokine storm management. However, comprehensive assurance of clinical safety and efficacy requires further investigation across several critical domains: (i) optimization of administration strategies (including route selection and dosing frequency), (ii) development of integrated efficacy assessment frameworks incorporating clinical outcomes, molecular biomarkers, and advanced imaging data, (iii) mechanistic elucidation of anti-aging effects at cellular and molecular levels, and (iv) implementation of large-scale multicenter clinical trials with extended (5–10 years) follow-up periods to assess long-term safety profiles and treatment durability.

MSC-derived exosomes (MSC-exosomes) have not been associated with the aforementioned safety concerns (e.g., carcinogenicity), owing to their ease of storage and transportation, high efficiency, and diverse sourcing. Consequently, they are regarded as promising substitutes for mesenchymal stem cells, fulfilling the criteria for cell-free therapeutic approaches (Li et al., 2016; Willis et al., 2018). Notably, they have demonstrated efficacy in animal models and wound healing (Ferreira et al., 2017; Deng et al., 2019), as well as in mitigating cellular premature aging (Tofino-Vian et al., 2017; Li et al., 2018). Organoids provide a robust platform for preclinical drug discovery, enabling efficient screening of candidate drugs before advancing to *in vivo* studies, thereby reducing drug development costs. Moreover, they hold potential for personalized medicine by modeling patient-specific drug responses, and for disease research by replicating key aspects of human organ function and pathology (Sun et al., 2020). Emerging platforms that integrate nanotechnology, CRISPR-mediated gene editing, and stem cell technology (Chou et al., 2020), are being leveraged to advance stem cell-based anti-aging research. For example, immune cells—including NK cells, macrophages, γδ T cells, and Treg cells—have shown considerable anti-aging potential (Qin et al., 2021; Pan et al., 2022). Innovative approaches, such as using targeted lipid nanoparticles (tLNPs) to deliver mRNA for *in vivo* generation of CAR-T cells, could significantly reduce manufacturing costs, shorten culture durations, and enhance safety. Furthermore, engineered iPSCs differentiated into innate immune cells (e.g., CAR-iMac and CAR-iNK cells) have been utilized to develop safe and effective cellular therapeutics.

We anticipate that with the continuous advancement of technology, cell therapy will increasingly be applied in the control of aging owing to the use of cutting-edge technologies such as gene editing and metabolomics. While aging remains an inevitable biological process, the ability to delay its onset and mitigate its effects is transforming the vision of healthy longevity into an achievable reality.
