# Supplementary material for: Recent progress in stem cell and immune cell-based interventions for aging and age-related disorders
Source: Front Aging. 2025 Jul 22;6:1638168. doi: 10.3389/fragi.2025.1638168 (PMC12321787; doi:10.3389/fragi.2025.1638168)
Supplement: Supplementary file 1 [file Table1.docx]

Supplementary Material

# Supplementary Tables

**Table 1.** **Ongoing clinical trials for frail elderly patients**

| **Cell types** | **Infusion pattern** | **Clinical stage** | **Identification number** | **Countries** | **Time frame** | **Number of studies** | **Result or state** |
| --- | --- | --- | --- | --- | --- | --- | --- |
| allogeneic BM-MSCs^a^ | vein | Phase I/II | NCT02065245 | University of Miami, USA | 1 year | 15/30 | Safe, Tolerant, Effective (Golpanian et al., 2016; Golpanian et al., 2017; Tompkins et al., 2017) |
| allogeneic BM-MSCs | vein | Phase IIb | NCT03169231 | University of Miami, USA | 6 months | 150 | Safe, Tolerant, Effective (Yousefi et al., 2022) |
| allogeneic BM-MSCs | vein | Phase I/II | NCT02982915 | University of Miami, USA | 1 year | 62 | completed |
| allogeneic BM-MSCs | vein | Phase II | jRCT2043200038 | National Gerontology Center, Japan | 6 months | 45 | recruitment |
| allogeneic UC-MSCs^b^ | vein | Phase I/II | NCT04314011 | Shanghai Oriental Hospital, China | 6 months | 30 | completed |
| allogeneic UC-MSCs^b^ | vein | Phase I/II | NCT04919135 | Vinmec Stem Cell and Gene Technology Institute, Viet Nam | 9 months | 5 | recruitment |
| allogeneic UC-MSCs^b^ | vein | Phase I | NCT05018767 | Science and Regenerative Medicine Foundation, Antigua and Barbuda | 4 years | 20 | recruitment |
| autologous AD-cSVF^c^ | vein | not apply | NCT03514537 | Healeon Medical Inc., USA | 6 months | 200 | Invited to register |
| allogeneic GMFFP^d^ | vein | Phase I/II | NCT03458429 | South Florida Bone Marrow/Stem Cell Transplantation Institute, USA | 3 years | 30 | Invited to register |
| autologous AD-hMSCs^e^  allogeneic AD/UC-hMSCs | vein | Phase I | NCT04174898 | Landmark Medical Center Limited, Malaysia | 1 year | 100 | unknown state |
| autologous NK cells | vein | not apply | ChiCTR-OOh-17011878 | Shanghai Long March Hospital, China | 4 weeks | 37 | NK function and phenotypic changes (Tang et al., 2022) |
| autologous NK cells | vein | not apply | not found | BioBox LLC, USA | 459 days | 5 | PBMC aging markers decreased (Chelyapov et al., 2022) |

^a^bone marrow-derived mesenchymal stem cells.

^b^umbilical cord mesenchymal stem cells.

^c^adipose-derived stem/stromal cellular stromal vascular fraction.

^d^granulocyte-colony stimulating factor.

^e^adipose tissue-derived human mesenchymal stem cells.

**Table 2. Ongoing clinical trials for skin rejuvenation**

| **Cell types** | **Infusion pattern** | **Clinical stage** | **Identification number** | **Countries** | **Time frame** | **Number of studies** | **Result or state** |
| --- | --- | --- | --- | --- | --- | --- | --- |
| autologous ADSCs^a^ | intradermal | unknown | unknown | Kobe University, Japan | 1 year | 8 | skin regeneration (Ichihashi et al., 2023) |
| allogeneic hUC-MSCs-CM^b^ | MN import | unknown | ChiCTR-INR-17013311 | Capital Medical University, China | 10 weeks | 28 | Safe, Tolerant, Effective (Liang et al., 2022) |
| autologous AD-SVF^c^ | intradermal | not apply | NCT02923219 | Affiliated Hospital of Xuzhou Medical University, China | 6 months | 50 | Safe and effective (Yin et al., 2020) |
| allogeneic AD-MSCs^d^  secretory body | MN or Laser | not apply | NCT05508191 | Indonesian University School of Medicine, Indonesia | 6 weeks | 30 | Dermoscopic appearance and total DPAS improvement (Yusharyahya et al., 2023) |
| allogeneic BM-MSCs^e^ | vein | Phase I/II | NCT01771679 | San Diego Military Medical Center, USA | 1 year | 29 | Active non-recruitment |
| autologous ADSCs^a^ | intradermal | not apply | NCT03928444 | Alexandria University, Egypt | 6 months | 15 | completed |
| allogeneic UC-MSCs^f^ exosome | intradermal | Phase I/II | NCT05813379 | Isfahan Medical University, Iran | 3 months | 20 | recruitment |
| autologous AD-SVF^c^ | intradermal | Phase I/II | IRCT20200127046282N31 | Tehran Medical University, Iran | 6 months | 30 | Recruitment completed |
| autologous AD-SVF^c^ | intradermal | Phase III | RPCEC00000362 | "Hermanos Ameijeiras" Surgical Clinical Hospital, Cuba | 1 year | 46 | recruitment |
| autologous AD-SVF^c^ | intradermal | Phase I | IRCT20200127046282N30 | Tehran Medical University, Iran | 6 months | 10 | Recruitment completed |

^a^ADSCs, adipose- derived stem cells.

^b^human umbilical cord mesenchymal stem cell conditioned culture medium.

^c^adipose-derived stromal vascular fraction.

^d^adipose tissue-derived mesenchymal stem cells.

^e^bone marrow-derived mesenchymal stem cells.

^f^umbilical cord mesenchymal stem cells.

**Table 3. Ongoing clinical trials in patients with neurodegenerative diseases**

| **Disease types** | **Cell types** | **Infusion pattern** | **Clinical stage** | **Identification number** | **Countries** | **Time frame** | **Number of studies** | **Result or state** |
| --- | --- | --- | --- | --- | --- | --- | --- | --- |
| AD^a^ | autologous AD-SVF^c^ | lateral ventricle | Phase I | unknown | California, United States | 3 years | 31 | Safe (Duma et al., 2019) |
| AD^a^ | allogeneic UC-MSCs^d^ | lateral ventricle | Phase I/IIa | NCT02054208 | Samsung Medical Center, Korea | 3 years | 9 | Safe, Tolerant, Effective (Kim et al., 2021) |
| AD^a^ | allogeneic BM-MSCs^e^ | vein | Phase I | NCT02600130 | University of California, National Institutes of Health, USA | 52 weeks | 33 | Safe, Tolerant, Effective (Brody et al., 2023) |
| AD^a^ | allogeneic UC-MSCs^d^ | lateral ventricle | Phase IIa | NCT03172117 | Samsung Medical Center, Korea | 3 years | 36 | completed |
| AD^a^ | allogeneic BM-MSCs^e^ | vein | Phase IIa | NCT05233774 | University of California, National Institutes of Health, USA | 41 weeks | 48 | Proactive, not recruiting |
| AD^a^ | autologous NK cells | vein | Phase I | NCT04678453 | Enken Biotech, Mexico | 6 months | 30 | Recruitment, partial results announced |
| PD^b^ | autologous BM-MSCs^e^ | vein / intranasal | unknown | unknown | Neurology and Neurosurgery, Belarus | 3 months | 23 | Effective, MDSUPDRS score, PDQ-39 scale, mood/sleep improvement (Boika et al., 2020) |
| PD^b^ | autologous midbrain dopaminergic progenitor cell | intracerebral nucleus | unknown | unknown | Massachusetts General Hospital, USA | 2 years | 1 | Safe, Tolerant, Effective (Schweitzer et al., 2020) |
| PD^b^ | autologous AD-SVF^c^ | Face, nose | Proof of concept pilot test | unknown | Wake Forest University Institute for Regenerative Medicine, USA | 5 years | 2 | Effective, qualitative improvement in motor and non-motor symptoms (Carstens et al., 2020) |
| PD^b^ | allogeneic BM-MSCs^e^ | vein | Phase I | NCT02611167 | University of Texas Health Sciences Center, USA | 1 year | 20 | Safe, Tolerant, Effective (Schiess et al., 2021) |
| PD^b^ | autologous ADSCs^f^ | vein | Phase I | jRCTb050190036 | Kyoto National Hospital, Japan | 6 months | 3 | Safe and effective (Shigematsu et al., 2022) |
| PD^b^ | autologous AD-MSCs^g^ | vein | Phase II | NCT04928287 | Hope Biological Stem Cell Research Foundation, USA | 52 weeks | 24 | completed |
| PD^b^ | mesencephalic dopaminergic neurons | intracerebral nucleus | Phase I | NCT04802733 | BlueRock Therapeutics, USA | 2 years | 12 | Proactive, no recruitment, partial results published (safe/tolerated/effective） |
| stroke | alloischemic tolerance BM-MSCs^e^ | vein | Phase I/II | NCT01297413 | University of California, USA | 1 year | 15/21 | Safe, Tolerant, Effective (Levy et al., 2019) |
| stroke | allogeneic human neural stem cells | intracerebral nucleus | unknown | unknown | Institute of Neuroscience and Psychology, University of Glasgow, UK | 1 year | 23 | Safe and effective (Muir et al., 2020) |
| stroke | allogeneic AD-MSCs^g^ | vein | Phase IIa | NCT01678534 | La Paz University Hospital, Spain | 2 years | 19 | Safe and effective (de Celis-Ruiz et al., 2022) |
| stroke | allogeneic AD-MSCs^g^ | vein | Phase IIb | NCT04280003 | La Paz University Hospital, Spain | 2 years | 30 | recruitment (de Celis-Ruiz et al., 2021) |
| stroke | allogeneic dental pulp stem cells | vein | Phase I/II | NCT04608838 | Teijin Pharmaceutical Co., Ltd. Japan | 91 days | 79 | completed (Suda et al., 2022) |
| stroke | autologous BM-MSCs^e^ | vein | Phase III | NCT01716481 | Samsung Medical Center, Korea | 90 days | 44 | Effective, motor function improved (Bang et al., 2022; Lee et al., 2022) |
| stroke | Placenta derived human neural stem cells | nasal cavity | Phase I | ChiCTR1900022741 | Renji Hospital, Shanghai Jiao Tong University School of Medicine, China | 6 months | 60 | recruitment |

^a^alzheimer's disease.

^b^Parkinson's disease.

^c^adipose-derived stromal vascular fraction.

^d^umbilical cord mesenchymal stem cells.

^e^bone marrow-derived mesenchymal stem cells.

^f^adipose- derived stem cells.

^g^adipose tissue-derived mesenchymal stem cells.

**Table 4. Ongoing clinical trials in patients with cardiovascular diseases**

| **Disease types** | **Cell types** | **Infusion pattern** | **Clinical stage** | **Identification number** | **Countries** | **Time frame** | **Number of studies** | **Result or state** |
| --- | --- | --- | --- | --- | --- | --- | --- | --- |
| AMI^a^ | autologous BM-MSCs^b^ | intramyocardial | Phase I | NCT00644410 | Copenhagen University Heart Centre, Denmark | 4 years | 60 | Safety, efficacy (prognosis) (Mathiasen et al., 2020) |
| AMI^a^ | allogeneic cardiac stem cells（CDCs） | of intracoronary | Phase II | NCT01458405 | Smidt Heart Institute, USA | 6 months | 134/124 | Safe and effective (Makkar et al., 2020; Ostovaneh et al., 2021) |
| AMI^a^ | autologous BM-MSCs^b^ | of intracoronary | Phase II/III | NCT04421274 | Sichuan Mental Health Center Hospital, PLA Naval General Hospital, China | 1 year | 43 | Safe (Zhang et al., 2021) |
| AMI^a^ | allogeneic BM-MPCs^c^ | transendo cardial | Phase III | NCT02032004 | Texas Heart Institute Clinical Research Center, USA | 66 months | 573 | Safe and effective (Perin et al., 2023) |
| heart failure | allogeneic UC-MSCs^d^ | vein | Phase I/II | NCT01739777 | Los Andes University School of Medicine, Chile | 1 year | 30 | Safe and effective (Bartolucci et al., 2017) |
| AMI^a^ | Autologous bone marrow-derived lineage negative stem/progenitor cells | of intracoronary | unknown | unknown | Medical University of Pomerania, Poland | 1 year | 34 | Safe and effective (Peregud-Pogorzelska et al., 2020) |
| AMI^a^ | allogeneic WJ-MSCs^e^ | of intracoronary | Phase III | NCT05043610 | Shiraz Medical University, Iran | 1 year | 240 | recruitment (Attar et al., 2022a) |
| AMI^a^ | allogeneic WJ-MSCs^e^ | of intracoronary | Phase II | IRCT202011160494008N1 | Iran | 6 months | 60 | recruitment (Attar et al., 2022b) |
| heart failure（IHF） | allogeneic CSCC_ASC^f^ | intramyocardial | Phase II | NCT02673164 | Hamburg Cardiology, Germany | 6 months | 138 | completed (Paitazoglou et al., 2019) |
| AMI^a^ | allogeneic CSCC_ASC^f^ | intramyocardial | Phase II | NCT03092284 | Copenhagen University Heart Centre, Denmark | 12 months | 81 | completed (Kastrup et al., 2017) |

^a^acute myocardial infarction.

^b^bone marrow-derived mesenchymal stem cells.

^c^bone marrow-derived mesenchymal precursor cells.

^d^umbilical cord mesenchymal stem cells.

^e^umbilical cord-derived Wharton’s jelly MSCs.

^f^adipose tissue-derived mesenchymal stromal cells.

**Table 5. Ongoing clinical trials for patients with autoimmune diseases**

| **Disease types** | **Cell types** | **Infusion pattern** | **Clinical stage** | **Identification number** | **Countries** | **Time frame** | **Number of studies** | **Result or state** |
| --- | --- | --- | --- | --- | --- | --- | --- | --- |
| RA^a^ | allogeneic WJ-MSCs^f^ | vein | Phase I/II | NCT01547091 | Cell Therapy Center, PLA Air Force 986 Hospital, China | 3 years | 64 | Safe and effective (Wang et al., 2019) |
| RA^a^ | autologous AD-MSCs^g^ | vein | Phase I/IIa | NCT03691909 | Hope Biosciences Stem Cell Research Foundation, USA | 52 weeks | 15 | Safe, Tolerant, Effective (Vij et al., 2022) |
| SLE^b^ | Autologous anti-CD19 CAR-T cells | vein | unknown | unknown | Alexandria University Erlangen, Germany | 1 year | 5 | Safe, Tolerant, Effective (Mackensen et al., 2022) |
| SLE^b^ | autologous anti-BCMA CAR-T cells | vein | Phase II | NCT06038474 | Cartesian Therapeutics, USA | 1 year | 30 | Not yet recruited |
| SLE^b^ | Autologous anti-CD19/CD20 CAR-T cells | vein | Phase I/II | NCT06153095 | ImmPACT Bio, University of California, USA | 1 year | 30 | Not yet recruited |
| SLE^b^ | Autologous anti-CD19 CAR-T cells | vein | Phase I | NCT05765006 | Shanghai Mingju Biotechnology Co., Ltd., China | 19 months | 24 | recruitment |
| SLE^b^ | Autologous anti-CD19 CAR-T cells | vein | Phase I | NCT05938725 | Kyverna Therapeutics, USA | 2 years | 12 | recruitment |
| SLE^b^ | Autologous anti-CD19 Nex-T CAR-T cells | vein | Phase I | NCT05869955 | Bristol-Myers Squibb, USA | 2 years | 43 | recruitment |
| SLE^b^ | Autologous anti-CD19 CAR-T cells | vein | Phase I/II | NCT06121297 | Cabaletta Bio, Massachusetts General Hospital, USA | 156 weeks | 12 | recruitment |
| SLE^b^ | Autologous anti-CD19 CAR-T cells | vein | Phase I/II | NCT05798117 | Novartis, USA | 2 years | 27 | recruitment |
| MG^c^ | autologous anti-BCMA CAR-T cells | vein | Phase IIb | NCT04146051 | Cartesian Therapeutics, USA | 168 days | 30 | recruitment (Granit et al., 2023) |
| ASS^d^ | Autologous anti-CD19 CAR-T cells | vein | unknown | unknown | German Immunotherapy Center, Germany | 6 months | 1 | Safe, Tolerant, Effective (Muller et al., 2023) |
| NMOSD^e^ | autologous anti-BCMA CAR-T cells | vein | Phase I | NCT04561557 | Huazhong University of Science and Technology, China | 14 months | 12 | Safe, Tolerant, Effective (Qin et al., 2023) |

^a^rheumatoid arthritis.

^b^systemic lupus erythematosus.

^c^Myasthenia gravis.

^d^anti-synthetase syndrome.

^e^Neuromyelitis optica spectrum disorder.

^f^umbilical cord-derived Wharton’s jelly MSCs.

^g^AD-MSCs, adipose tissue-derived mesenchymal stem cells.

References

Attar, A., Monabati, A., Montaseri, M., Vosough, M., Hosseini, S.A., Kojouri, J., et al. (2022a). Transplantation of mesenchymal stem cells for prevention of acute myocardial infarction induced heart failure: study protocol of a phase III randomized clinical trial (Prevent-TAHA8). Trials 23(1), 632. doi: 10.1186/s13063-022-06594-1.

Attar, A., Nouri, F., Yazdanshenas, A., Hessami, K., Vosough, M., Abdi-Ardekani, A., et al. (2022b). Single vs. double intracoronary injection of mesenchymal stromal cell after acute myocardial infarction: the study protocol from a randomized clinical trial: BOOSTER-TAHA7 trial. Trials 23(1), 293. doi: 10.1186/s13063-022-06276-y.

Bang, O.Y., Kim, E.H., Cho, Y.H., Oh, M.J., Chung, J.W., Chang, W.H., et al. (2022). Circulating Extracellular Vesicles in Stroke Patients Treated With Mesenchymal Stem Cells: A Biomarker Analysis of a Randomized Trial. Stroke 53(7), 2276-2286. doi: 10.1161/STROKEAHA.121.036545.

Bartolucci, J., Verdugo, F.J., Gonzalez, P.L., Larrea, R.E., Abarzua, E., Goset, C., et al. (2017). Safety and Efficacy of the Intravenous Infusion of Umbilical Cord Mesenchymal Stem Cells in Patients With Heart Failure: A Phase 1/2 Randomized Controlled Trial (RIMECARD Trial [Randomized Clinical Trial of Intravenous Infusion Umbilical Cord Mesenchymal Stem Cells on Cardiopathy]). Circ Res 121(10), 1192-1204. doi: 10.1161/CIRCRESAHA.117.310712.

Boika, A., Aleinikava, N., Chyzhyk, V., Zafranskaya, M., Nizheharodava, D., and Ponomarev, V. (2020). Mesenchymal stem cells in Parkinson's disease: Motor and nonmotor symptoms in the early posttransplant period. Surg Neurol Int 11, 380. doi: 10.25259/SNI_233_2020.

Brody, M., Agronin, M., Herskowitz, B.J., Bookheimer, S.Y., Small, G.W., Hitchinson, B., et al. (2023). Results and insights from a phase I clinical trial of Lomecel-B for Alzheimer's disease. Alzheimers Dement 19(1), 261-273. doi: 10.1002/alz.12651.

Carstens, M., Haq, I., Martinez-Cerrato, J., Dos-Anjos, S., Bertram, K., and Correa, D. (2020). Sustained clinical improvement of Parkinson's disease in two patients with facially-transplanted adipose-derived stromal vascular fraction cells. J Clin Neurosci 81, 47-51. doi: 10.1016/j.jocn.2020.09.001.

Chelyapov, N., Nguyen, T.T., and Gonzalez, R. (2022). Autologous NK cells propagated and activated ex vivo decrease senescence markers in human PBMCs. Biochem Biophys Rep 32, 101380. doi: 10.1016/j.bbrep.2022.101380.

de Celis-Ruiz, E., Fuentes, B., Alonso de Lecinana, M., Gutierrez-Fernandez, M., Borobia, A.M., Gutierrez-Zuniga, R., et al. (2022). Final Results of Allogeneic Adipose Tissue-Derived Mesenchymal Stem Cells in Acute Ischemic Stroke (AMASCIS): A Phase II, Randomized, Double-Blind, Placebo-Controlled, Single-Center, Pilot Clinical Trial. Cell Transplant 31, 9636897221083863. doi: 10.1177/09636897221083863.

de Celis-Ruiz, E., Fuentes, B., Moniche, F., Montaner, J., Borobia, A.M., Gutierrez-Fernandez, M., et al. (2021). Allogeneic adipose tissue-derived mesenchymal stem cells in ischaemic stroke (AMASCIS-02): a phase IIb, multicentre, double-blind, placebo-controlled clinical trial protocol. BMJ Open 11(8), e051790. doi: 10.1136/bmjopen-2021-051790.

Duma, C., Kopyov, O., Kopyov, A., Berman, M., Lander, E., Elam, M., et al. (2019). Human intracerebroventricular (ICV) injection of autologous, non-engineered, adipose-derived stromal vascular fraction (ADSVF) for neurodegenerative disorders: results of a 3-year phase 1 study of 113 injections in 31 patients. Mol Biol Rep 46(5), 5257-5272. doi: 10.1007/s11033-019-04983-5.

Golpanian, S., DiFede, D.L., Khan, A., Schulman, I.H., Landin, A.M., Tompkins, B.A., et al. (2017). Allogeneic Human Mesenchymal Stem Cell Infusions for Aging Frailty. J Gerontol A Biol Sci Med Sci 72(11), 1505-1512. doi: 10.1093/gerona/glx056.

Golpanian, S., DiFede, D.L., Pujol, M.V., Lowery, M.H., Levis-Dusseau, S., Goldstein, B.J., et al. (2016). Rationale and design of the allogeneiC human mesenchymal stem cells (hMSC) in patients with aging fRAilTy via intravenoUS delivery (CRATUS) study: A phase I/II, randomized, blinded and placebo controlled trial to evaluate the safety and potential efficacy of allogeneic human mesenchymal stem cell infusion in patients with aging frailty. Oncotarget 7(11), 11899-11912. doi: 10.18632/oncotarget.7727.

Granit, V., Benatar, M., Kurtoglu, M., Miljkovic, M.D., Chahin, N., Sahagian, G., et al. (2023). Safety and clinical activity of autologous RNA chimeric antigen receptor T-cell therapy in myasthenia gravis (MG-001): a prospective, multicentre, open-label, non-randomised phase 1b/2a study. Lancet Neurol 22(7), 578-590. doi: 10.1016/S1474-4422(23)00194-1.

Ichihashi, M., Tanaka, M., Iizuka, T., Totsuka, H., Tominaga, E., Hitomi, Y., et al. (2023). A Single Intradermal Injection of Autologous Adipose-Tissue-Derived Stem Cells Rejuvenates Aged Skin and Sharpens Double Eyelids. J Pers Med 13(7). doi: 10.3390/jpm13071162.

Kastrup, J., Schou, M., Gustafsson, I., Nielsen, O.W., Mogelvang, R., Kofoed, K.F., et al. (2017). Rationale and Design of the First Double-Blind, Placebo-Controlled Trial with Allogeneic Adipose Tissue-Derived Stromal Cell Therapy in Patients with Ischemic Heart Failure: A Phase II Danish Multicentre Study. Stem Cells Int 2017, 8506370. doi: 10.1155/2017/8506370.

Kim, H.J., Cho, K.R., Jang, H., Lee, N.K., Jung, Y.H., Kim, J.P., et al. (2021). Intracerebroventricular injection of human umbilical cord blood mesenchymal stem cells in patients with Alzheimer's disease dementia: a phase I clinical trial. Alzheimers Res Ther 13(1), 154. doi: 10.1186/s13195-021-00897-2.

Lee, J., Chang, W.H., Chung, J.W., Kim, S.J., Kim, S.K., Lee, J.S., et al. (2022). Efficacy of Intravenous Mesenchymal Stem Cells for Motor Recovery After Ischemic Stroke: A Neuroimaging Study. Stroke 53(1), 20-28. doi: 10.1161/STROKEAHA.121.034505.

Levy, M.L., Crawford, J.R., Dib, N., Verkh, L., Tankovich, N., and Cramer, S.C. (2019). Phase I/II Study of Safety and Preliminary Efficacy of Intravenous Allogeneic Mesenchymal Stem Cells in Chronic Stroke. Stroke 50(10), 2835-2841. doi: 10.1161/STROKEAHA.119.026318.

Liang, X., Li, J., Yan, Y., Xu, Y., Wang, X., Wu, H., et al. (2022). Efficacy of Microneedling Combined With Local Application of Human Umbilical Cord-Derived Mesenchymal Stem Cells Conditioned Media in Skin Brightness and Rejuvenation: A Randomized Controlled Split-Face Study. Front Med (Lausanne) 9, 837332. doi: 10.3389/fmed.2022.837332.

Mackensen, A., Muller, F., Mougiakakos, D., Boltz, S., Wilhelm, A., Aigner, M., et al. (2022). Anti-CD19 CAR T cell therapy for refractory systemic lupus erythematosus. Nat Med 28(10), 2124-2132. doi: 10.1038/s41591-022-02017-5.

Makkar, R.R., Kereiakes, D.J., Aguirre, F., Kowalchuk, G., Chakravarty, T., Malliaras, K., et al. (2020). Intracoronary ALLogeneic heart STem cells to Achieve myocardial Regeneration (ALLSTAR): a randomized, placebo-controlled, double-blinded trial. Eur Heart J 41(36), 3451-3458. doi: 10.1093/eurheartj/ehaa541.

Mathiasen, A.B., Qayyum, A.A., Jorgensen, E., Helqvist, S., Kofoed, K.F., Haack-Sorensen, M., et al. (2020). Bone marrow-derived mesenchymal stromal cell treatment in patients with ischaemic heart failure: final 4-year follow-up of the MSC-HF trial. Eur J Heart Fail 22(5), 884-892. doi: 10.1002/ejhf.1700.

Muir, K.W., Bulters, D., Willmot, M., Sprigg, N., Dixit, A., Ward, N., et al. (2020). Intracerebral implantation of human neural stem cells and motor recovery after stroke: multicentre prospective single-arm study (PISCES-2). J Neurol Neurosurg Psychiatry 91(4), 396-401. doi: 10.1136/jnnp-2019-322515.

Muller, F., Boeltz, S., Knitza, J., Aigner, M., Volkl, S., Kharboutli, S., et al. (2023). CD19-targeted CAR T cells in refractory antisynthetase syndrome. Lancet 401(10379), 815-818. doi: 10.1016/S0140-6736(23)00023-5.

Ostovaneh, M.R., Makkar, R.R., Ambale-Venkatesh, B., Ascheim, D., Chakravarty, T., Henry, T.D., et al. (2021). Effect of cardiosphere-derived cells on segmental myocardial function after myocardial infarction: ALLSTAR randomised clinical trial. Open Heart 8(2). doi: 10.1136/openhrt-2021-001614.

Paitazoglou, C., Bergmann, M.W., Vrtovec, B., Chamuleau, S.A.J., van Klarenbosch, B., Wojakowski, W., et al. (2019). Rationale and design of the European multicentre study on Stem Cell therapy in IschEmic Non-treatable Cardiac diseasE (SCIENCE). Eur J Heart Fail 21(8), 1032-1041. doi: 10.1002/ejhf.1412.

Peregud-Pogorzelska, M., Przybycien, K., Baumert, B., Kotowski, M., Pius-Sadowska, E., Safranow, K., et al. (2020). The Effect of Intracoronary Infusion of Autologous Bone Marrow-Derived Lineage-Negative Stem/Progenitor Cells on Remodeling of Post-Infarcted Heart in Patient with Acute Myocardial Infarction. Int J Med Sci 17(8), 985-994. doi: 10.7150/ijms.42561.

Perin, E.C., Borow, K.M., Henry, T.D., Mendelsohn, F.O., Miller, L.W., Swiggum, E., et al. (2023). Randomized Trial of Targeted Transendocardial Mesenchymal Precursor Cell Therapy in Patients With Heart Failure. J Am Coll Cardiol 81(9), 849-863. doi: 10.1016/j.jacc.2022.11.061.

Qin, C., Tian, D.S., Zhou, L.Q., Shang, K., Huang, L., Dong, M.H., et al. (2023). Anti-BCMA CAR T-cell therapy CT103A in relapsed or refractory AQP4-IgG seropositive neuromyelitis optica spectrum disorders: phase 1 trial interim results. Signal Transduct Target Ther 8(1), 5. doi: 10.1038/s41392-022-01278-3.

Schiess, M., Suescun, J., Doursout, M.F., Adams, C., Green, C., Saltarrelli, J.G., et al. (2021). Allogeneic Bone Marrow-Derived Mesenchymal Stem Cell Safety in Idiopathic Parkinson's Disease. Mov Disord 36(8), 1825-1834. doi: 10.1002/mds.28582.

Schweitzer, J.S., Song, B., Herrington, T.M., Park, T.Y., Lee, N., Ko, S., et al. (2020). Personalized iPSC-Derived Dopamine Progenitor Cells for Parkinson's Disease. N Engl J Med 382(20), 1926-1932. doi: 10.1056/NEJMoa1915872.

Shigematsu, K., Komori, N., Tahara, K., and Yamagishi, H. (2022). Repeated infusion of autologous adipose tissue-derived stem cells for Parkinson's disease. Acta Neurol Scand 145(1), 119-122. doi: 10.1111/ane.13547.

Suda, S., Nito, C., Ihara, M., Iguchi, Y., Urabe, T., Matsumaru, Y., et al. (2022). Randomised placebo-controlled multicentre trial to evaluate the efficacy and safety of JTR-161, allogeneic human dental pulp stem cells, in patients with Acute Ischaemic stRoke (J-REPAIR). BMJ Open 12(5), e054269. doi: 10.1136/bmjopen-2021-054269.

Tang, X., Deng, B., Zang, A., He, X., Zhou, Y., Wang, D., et al. (2022). Characterization of age-related immune features after autologous NK cell infusion: Protocol for an open-label and randomized controlled trial. Front Immunol 13, 940577. doi: 10.3389/fimmu.2022.940577.

Tompkins, B.A., DiFede, D.L., Khan, A., Landin, A.M., Schulman, I.H., Pujol, M.V., et al. (2017). Allogeneic Mesenchymal Stem Cells Ameliorate Aging Frailty: A Phase II Randomized, Double-Blind, Placebo-Controlled Clinical Trial. J Gerontol A Biol Sci Med Sci 72(11), 1513-1522. doi: 10.1093/gerona/glx137.

Vij, R., Stebbings, K.A., Kim, H., Park, H., and Chang, D. (2022). Safety and efficacy of autologous, adipose-derived mesenchymal stem cells in patients with rheumatoid arthritis: a phase I/IIa, open-label, non-randomized pilot trial. Stem Cell Res Ther 13(1), 88. doi: 10.1186/s13287-022-02763-w.

Wang, L., Huang, S., Li, S., Li, M., Shi, J., Bai, W., et al. (2019). Efficacy and Safety of Umbilical Cord Mesenchymal Stem Cell Therapy for Rheumatoid Arthritis Patients: A Prospective Phase I/II Study. Drug Des Devel Ther 13, 4331-4340. doi: 10.2147/DDDT.S225613.

Yin, Y., Li, J., Li, Q., Zhang, A., and Jin, P. (2020). Autologous fat graft assisted by stromal vascular fraction improves facial skin quality: A randomized controlled trial. J Plast Reconstr Aesthet Surg 73(6), 1166-1173. doi: 10.1016/j.bjps.2019.11.010.

Yousefi, K., Ramdas, K.N., Ruiz, J.G., Walston, J., Arai, H., Volpi, E., et al. (2022). The Design and Rationale of a Phase 2b, Randomized, Double-Blinded, and Placebo-Controlled Trial to Evaluate the Safety and Efficacy of Lomecel-B in Older Adults with Frailty. J Frailty Aging 11(2), 214-223. doi: 10.14283/jfa.2022.2.

Yusharyahya, S.N., Japranata, V.V., Sitohang, I.B.S., Legiawati, L., Novianto, E., Suseno, L.S., et al. (2023). A Comparative Study on Adipose-Derived Mesenchymal Stem Cells Secretome Delivery Using Microneedling and Fractional CO(2) Laser for Facial Skin Rejuvenation [Response to Letter]. Clin Cosmet Investig Dermatol 16, 671-672. doi: 10.2147/CCID.S410433.

Zhang, R., Yu, J., Zhang, N., Li, W., Wang, J., Cai, G., et al. (2021). Bone marrow mesenchymal stem cells transfer in patients with ST-segment elevation myocardial infarction: single-blind, multicenter, randomized controlled trial. Stem Cell Res Ther 12(1), 33. doi: 10.1186/s13287-020-02096-6.
